# Comparison of melatonin and curcumin effect at the light and dark periods on regeneration of sciatic nerve crush injury in rats

**DOI:** 10.17179/excli2019-1369

**Published:** 2019-08-21

**Authors:** Farshad Moharrami Kasmaie, Zohreh Jahromi, Rouhollah Gazor, Arash Zaminy

**Affiliations:** 1 Student Research Committee, School of Medicine, Guilan University of Medical Sciences, Rasht, Iran; 2 Department of Anatomical Sciences, School of Medicine, Guilan University of Medical Sciences, Rasht, Iran; 3 Neuroscience Research Center, Guilan University of Medical Sciences, Rasht, Iran

**Keywords:** melatonin, curcumin, nerve injury, anti-inflammatory, antioxidant, crush

## Abstract

Being one of the acute clinical problems, peripheral nerve injury can bring about a number of consequences including severe disability, reduced Quality of life (QOL) and immense costs. Currently, melatonin and curcumin are widely applied because of their immunomodulatory, anti-inflammatory, neuro-protective and antioxidant properties. The present study aims to compare the effects of melatonin and curcumin during light and dark periods on sciatic nerve crush injury repair. Accordingly, rats received IP injections of curcumin (100 mg/kg) and melatonin (10 mg/kg) over two periods of light (9:00 a.m.) and dark (9:00 p.m.) for 4 weeks. In order to evaluate rats, functional (walking track analysis and electrophysiological measurements), histomorphometric and gastrocnemius muscle mass investigations were administered. No statistically significant difference was identified between dark and light curcumin groups while curcumin groups displayed better results than did melatonin groups. In addition, dark melatonin group displayed better results than the light melatonin. On the whole, this study found that melatonin and curcumin can be used to quicken neural recovery and help treat nerve injury. It was also found that better neuroregeneration or nerve regeneration was induced when rats were treated by melatonin during the dark period while effects and injection time did not correlate in curcumin application.

## Introduction

As a common clinical problem, peripheral nerve injury is caused by a variety of factors such as trauma. As a result, many people suffer these injuries every year. These injuries cause a number of problems including severe disability, reduced QOL and immense costs (Fatemi et al., 2016[10]; Jones et al., 2016[14]). The etiological identification of the affected lesions is highly diverse (it can include elongation, section, laceration, crushing) (Caillaud et al., 2018[4]). Consequently, any erroneous or improper treatment can lead to loss of sensory, motor and autonomous functions (Yüce et al., 2015[45]). Approximately, 24-48 hours after the neuronal damage, the Wallerian degeneration sets in, a series of cellular and molecular changes. This process sets off some inflammatory and chemical reactions, death of dorsal root ganglions (DRGs), comprehensive cell migration and proliferation, death of spinal cord neurons, as well as apoptosis. In turn, these events lead to free radicals, formation, and accumulation of toxic substances, as well as scar tissue that themselves disrupt axonal regrowth and repair (Houschyar et al., 2016[12]; Faroni et al., 2015[9]; Atik et al., 2011[2]; Ma et al., 2016[22]). Although significant progress has been made in the pathophysiological understanding of peripheral nerve injury and repair and despite the empirical studies on minimizing the results of neural damages, neural recovery and regrowth still remain a major challenge (Yüce et al., 2015[45]; Houschyar et al., 2016[12]). Over the recent years, many studies have addressed and evaluated various beneficial treatments such as administration of biological and chemical drugs, hormones, neurotrophic growth factors, cell therapy and so on (Yüce et al., 2015[45]; Panagopoulos et al., 2017[32]). It has been shown that prescribing certain drugs helps nerve tissues recover and repair. Commonly used drugs include tacrolimus, hyaluronic acid, melatonin, curcumin and methyl prednisolone (Ma et al., 2016[22]; Mekaj and Mekaj, 2017[25]).

Made from the powdered rhizome of turmeric (Curcuma longa), curcumin is a yellowish, multi-potent and biologically active chemical that has been used in traditional medicine for centuries with no toxicity reports until the present. Curcumin is used in food, pharmaceuticals, cosmetics, and medicine (for its chemotherapy properties) and is also known to possess healing effects for a variety of diseases including inflammatory conditions (Yüce et al., 2015[45]; Mazaheri et al., 2017[24]). Curcumin possesses excellent neuro-protective, antioxidant, anti-apoptotic, anti-inflammatory properties and can balance enzymes and inflammatory cytokines (Yüce et al., 2015[45]; Liu et al., 2016[21]; Mazaheri et al., 2017[24]; Noorafshan et al., 2011[28]; Mohammadi and Mahmoodi, 2013[27]). Curcumin can be used to protect the brain against ischemia, heal spinal cord injury, and quicken damaged peripheral nerve repair as it can strengthen central and peripheral nervous systems (Ma et al., 2016[22]; Mekaj and Mekaj, 2017[25]). 

As another commonly used drug in healing nerve damage, melatonin (N-acetyl methoxy-tryptamine) is the primary hormone that is secreted from the pineal gland in all creatures at night. Melatonin plays a role in circadian rhythm regulation and also in the physiology of sleep, mental activities, reproduction, etc. (Atik et al., 2011[2]; Turgut and Kaplan, 2011[41]). As a potent free radical scavenger, melatonin has many properties like neuroprotective effect, antioxidant, anti-inflammatory immunomodulation and also protective of macromolecules (e.g. DNA) against oxidative damage (Turgut and Kaplan, 2011[41]; Kaplan et al., 2011[15]; Kaya et al., 2013[16][17]). Melatonin is also known to have a positive effect on the content of myelin and the number of axons by decreasing collagen accumulation, preventing the formation of neuroma and scar tissue on nerve damage site and stimulating the Schwann cells proliferation (Chang et al., 2014[6]; Onger et al., 2017[31]). The medical function of melatonin is subject to various factors including temperature, humidity, circadian rhythm and also storage duration in medical institutions and at households (Yamashita et al., 2018[43]). Noticeable progress has been made in the cellular and molecular understanding of circadian rhythm over the last two decades (Dibner and Schibler, 2015[8]). Circadian rhythm is defined as a 24-hour endogenous oscillation associated with the behavioral and physiological changes of mammals which can adapt their physiological activities during night and day thanks to this internal clock (Zhang et al., 2014[47]). Studies have indicated that the effects of some drugs including melatonin can differ during periods of light and darkness (Rateb et al., 2017[34]). Several reports indicate the positive effects of melatonin and curcumin on nerve damage repair; so, this study intends to comparatively evaluate melatonin and curcumin at light and dark periods in terms of their effects on the regeneration of sciatic nerve crush injury in rats.

## Materials and Methods

### Animals and experimental groups 

This study involved fifty-six male Wistar rats weighing between 250-300 g. The entire research and animal care procedures were approved by the Ethics Committee of Guilan University of Medical Sciences. All animals were kept in standard plastic cages with free access to food and water under these stable laboratory conditions: ambient temperature (18 °C to 21 °C), humidity (55 % ± 5 %), and 12-h light-dark cycles.

Rats were randomly assigned to seven groups (n=8): Sham (control), curcumin during dark period (D Cur), Vehicle (DMSO), curcumin during light period (L Cur), melatonin during light period (L Mel), crush (injury without any intervention), and melatonin during dark period (D Mel).

### Surgical procedures

Anesthesia was induced by IP injections of ketamine (100 mg/kg) and xylazine (10 mg/kg). Animals were shaved after anesthesia and skin region was disinfected with an antiseptic solution. Subsequently, an incision was used to open the skin and fascia of the posterior part of the left thigh for about 2 cm. Subsequently, the vastus lateralis and biceps femoris muscles were removed apart so that the sciatic nerve could be exposed. In experimental groups, the sciatic nerve was crushed at a point 1 cm proximal to the bifurcation point. A clamper was used to perform crush injury for about 2 minutes. Using a 4/0 chromic gut suture, the muscle layers were re-connected while 4/0 silk sutures were applied to close the skin. After surgery, the rats were monitored during their recovery from the anesthetic state under heating lamps and were subsequently left to feed and drink *ad libitum* (Chauderlier et al., 2017[7]; Li et al., 2017[20]).

### Drug administration

Curcumin, melatonin and dimethyl sulfoxide (DMSO) were bought from Sigma-Aldrich. Curcumin and melatonin were dissolved in DMSO and diluted with distilled water. The final DMSO concentration was reached to be 5 %. DMSO (5 % in distilled water) was administered for the vehicle group. For 4 weeks after surgery, each rat received IP injections of curcumin 100 mg/kg and melatonin 10 mg/kg during two periods of light (9:00 a.m.) and darkness (9:00 p.m.) (Wang et al., 2015[42]; Chamanara et al., 2019[5]).

### Sciatic functional index (SFI)

Sciatic functional index (SFI) was performed on days 1, 7, 14, 21, and 28 following the surgery. As a part of this test, the rats' hind paws were marked by black ink and the animal models were immediately freed to walk along a path (7.5*50 cm^2^). Subsequently, the activity index of the sciatic nerve was measured using the footprint of rats on a white paper and also by the formula of Bain et al. (1989). According to this procedure, SFI value of -100 shows significant impairment whereas an SFI value of around 0 is indicative of normal function (Jeong et al., 2017[13]).

### Electrophysiological analysis

On the 28^th^ day after the surgical operation, all groups underwent electrophysiology. At this point, the anesthetic state was induced and the left sciatic nerve was exposed to assess motor functional recovery. The amplitudes and conduction latencies of the evoked compound muscle action potential (CMAP) were recorded in the gastrocnemius muscle (E-Wave, Science beam, Iran). The ranges of stimulation intensity and filtration were 1000 mA and 0.2 Hz, respectively.

### Gastrocnemius muscle mass measurement

The weight ratio of the gastrocnemius muscle was used in order to perform a recovery assessment. Right after sacrificing the rats, gastrocnemius muscles were carefully dissected and harvested from both intact (non-operative) and injured (operative) sides and weighed while they were still wet. In order to determine the weight ratios (in percentages), the weights of muscle mass from the injured sides were divided by those from the normal sides (Schiraldi et al., 2018[39]).

### Histological evaluations

For histological and immunohistochemical assessments, the injured segments of the sciatic nerve and a middle third of the gastrocnemius muscle were put in 10 % formaldehyde solution in order to fix. Once the tissues were processed, paraffin was used to embed samples. Subsequently, paraffin blocks were divided at 5 µm intervals. After deparaffinization and rehydration processes, tissue sections were prepared for staining. To evaluate histological stains, hematoxylin-eosin staining (H&E) was used to show the morphology of the gastrocnemius muscle and to determine the diameter of muscle fibers. Additionally, myelin content was evaluated by Luxol Fast Blue (LFB) staining. Specific proteins expression patterns were detected using Immunohistochemical (IHC) staining. In order to determine the number of Schwann cells and axon neurofilaments, rabbit anti-S100 (1:100 dilution, Abcam), and rabbit anti-neurofilament-200 (NF-200, 1:100 dilution, Abcam) monoclonal antibodies, respectively, were used as primary antibodies overnight at 4 °C. Subsequently, the sections were incubated with goat HRP-conjugated anti-rabbit secondary antibody (1:1000 dilution, Abcam) at room temperature for 1 hour. Diaminobenzidine (DAB) (SIGMAFAST™ DAB with Metal Enhancer, Sigma) was applied as the chromogen. A light microscope was used to examine the sections. Five sections for each rat at equal intervals were selected for quantification. In the case of each section, all positive areas in five random microscopic high-power fields (400x) were analyzed and averaged. ImageJ (NIH, Bethesda, MD) was used to measure LFB color intensity, number of Schwann cells and axon neurofilaments while Digimizer (v 4.3.0.) software was used to measure the diameter of muscles fibers.

### Total Oxidant Status (TOS)

Total Oxidant Assay kit (Kiazist Company, Tehran, Iran) was used to determine TOS. The total amount of oxidants for all serum samples was checked which consisted of reactive oxygen species (ROS) or reactive nitrogen species (RNS). During this test, Ferrous iron (Fe^2+^) is oxidized in Ferric form (Fe^3+^) in the presence of oxidants and stained by chromogen. This color was measured through spectrophotometry at a wavelength of 570 nm.

### Statistical analysis

In the present study, all data are expressed as mean ± Standard Deviation (SD). Kolmogorov-Smirnov test was used for evaluating normality. One-way analyses of variance (ANOVAs) was used in order to compare the mean values of the groups. Graphpad Prism 7.04 software was used to do all the statistical analyses. The probability was considered as significant when p <0.05.

## Results

### SFI 

**Figure 1:**
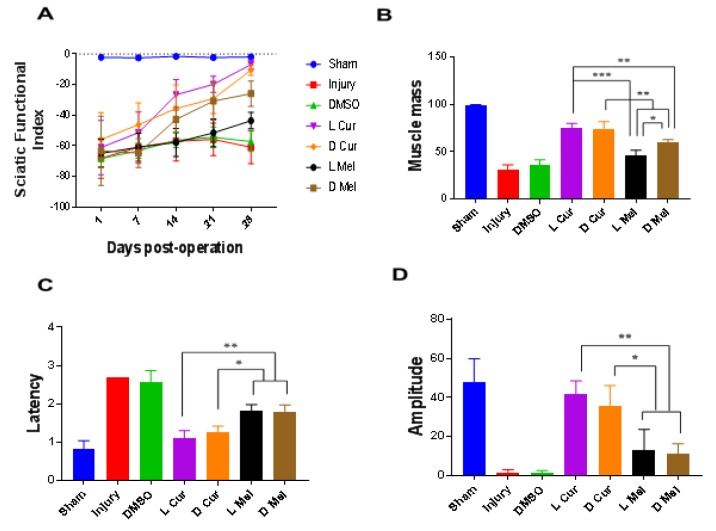
Functional evaluations. Walking track analysis and electrophysiological measurements and muscle weight measurement at the end of the 28^th^ day. (A) The SFI values were assessed on the first day after surgery and on days 7, 14, 21, and 28 in the sham, injury, DMSO and treatment groups. In the treatment groups, no significant difference was observed between L Cur and D Cur groups. Moreover, in both Cur groups, the SFI value was better than in Mel groups. (B) Gastrocnemius muscle ratios (experimental side/normal side) were calculated in the fourth week in all groups. L Cur and D Cur groups showed no significant difference. The Cur groups displayed the lowest amount of atrophy and proved better than Mel groups. The dark melatonin group also displayed better results compared with the light melatonin group. (C&D) The latency time and amplitude values were evaluated between groups at the end of the 4^th^ week. The L Cur and D Cur groups showed no significant difference and, compared with other groups, they had the least latency and the greatest amplitude (P < 0.05). * P < 0.05, ** P < 0.01, *** P < 0.001.

Using SFI data on the 28^th^ post-operation day, it was shown that motor functional recovery was better in the treatment groups than in the injury and DMSO groups. In the case of treatment groups, no significant difference was observed between L Cur and D Cur groups. Moreover, in both Cur groups, the SFI value was found to be greater than in Mel groups. The melatonin groups, D Mel group showed a significant difference compared with L Mel groups. No statistically significant difference was observed between the injury and DMSO groups while the SFI value was the smallest. The SFI value in L Mel group was improved compared with injury and DMSO groups; however, no significant difference was observed between L Mel group and injury groups (Figure 1A(Fig. 1)).

### Gastrocnemius muscle mass

The gastrocnemius muscle mass ratio was measured and compared in the case of both damaged foot and healthy foot muscle for all rats. A statistically significant difference was observed between sham, injury, vehicle and treatment groups. The sham group displayed the best result among other groups and no muscle atrophy was detected. In the case of treatment groups, L Cur and D Cur groups showed no significant difference. The Cur groups displayed the smallest extent of atrophy and proved better than Mel groups. The dark melatonin group also displayed better results compared with the light melatonin group. The muscle weight for L Mel group was greater than the muscle weight for injury and DMSO groups but proved not significantly different than the DMSO group. DMSO and injury groups showed no difference and had the largest atrophy in comparison with the treatment groups (Figure 1B(Fig. 1)).

### Electrophysiological test 

On the 28^th^ post-operation day, motor functional recovery was assessed and evaluated using an electrophysiological test. The onset latency and peak amplitude of CMAP were analyzed for all groups. The L Cur and D Cur groups showed no significant difference and, compared with other groups, they showed the shortest latency and the greatest amplitude (Figure 1C-D(Fig. 1)).

### Total Oxidant Status (TOS)

**Figure 2:**
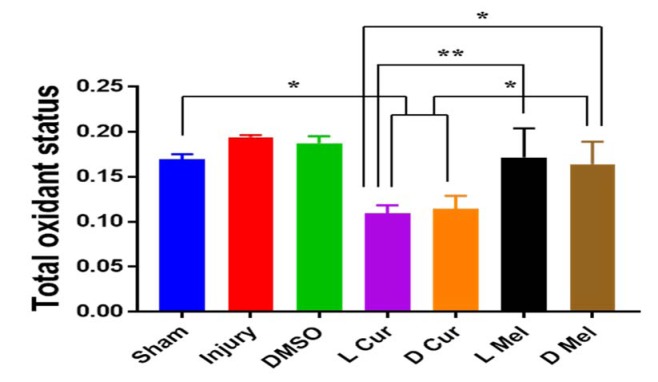
Total oxidant status. Serum TOS levels were measured in Sham, Injury, DMSO, and Treatment groups. No statistically significant difference was found between L and D melatonin groups. In curcumin groups, TOS was significantly lesser than Melatonin groups. * P < 0.05, ** P < 0.01.

Serum TOS level was determined for each experimental group. In the case of treatment groups, TOS in serum samples was significantly lesser than other groups. No statistically significant difference was found between L and D melatonin groups. In the case of curcumin groups, TOS was significantly lesser than melatonin groups (Figure 2(Fig. 2)).

### Histological evaluations

**Figure 3:**
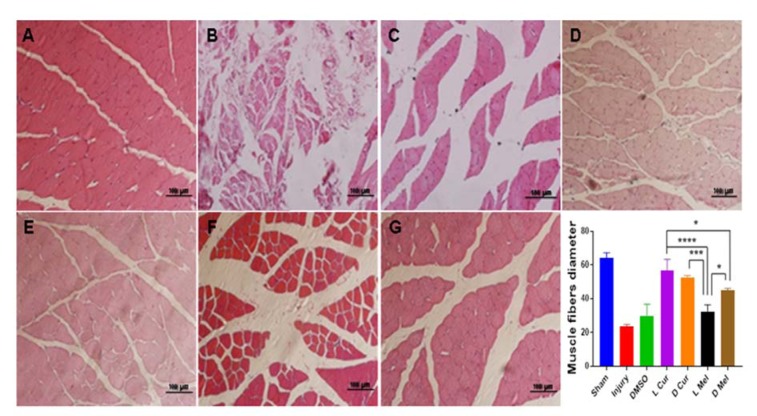
Representative light micrographs of gastrocnemius muscle fibers 4 weeks after injury. Hematoxylin-eosin staining (H&E) was used to show the gastrocnemius muscle morphology and to measure the diameter of the fibers. The sham group (A) displayed the best result among injury (B), DMSO (C), and experimental groups and no muscle atrophy was detected no significant difference was found between L Cur (D) and D Cur (E) groups while Curcumin groups displayed better results compared with Melatonin groups. In Melatonin groups, data showed that administering Melatonin in the dark period (G) left a stronger effect than the light period (F). * P < 0.05, *** P < 0.001, **** P < 0.0001. Scale bar: 100 µm.

**Figure 4:**
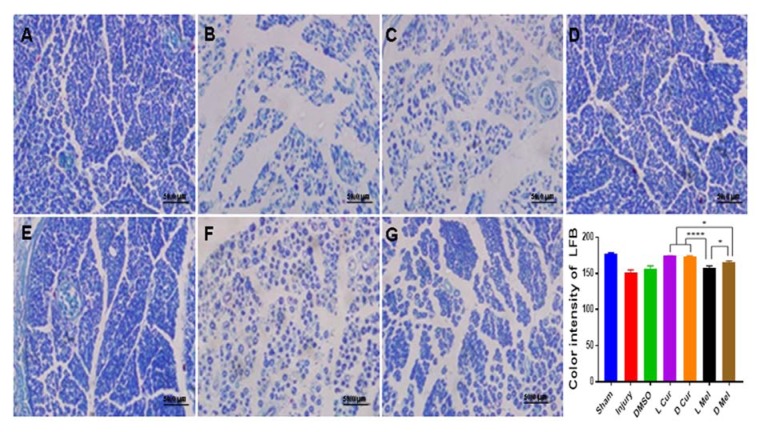
Luxol fast blue staining. The staining was used to evaluate myelination, and then all groups were compared in terms of color intensity of staining. The color intensity of the sham group (A) was found to be higher than injury (B), DMSO (C), Cur, and Mel groups. In treatment groups, L Cur (D) and D Cur (E) groups showed no statistically significant difference. The D Mel (G) group proved to be significantly higher than L Mel (F) group. * P < 0.05, **** P < 0.0001. Scale bar: 50 µm.

**Figure 5:**
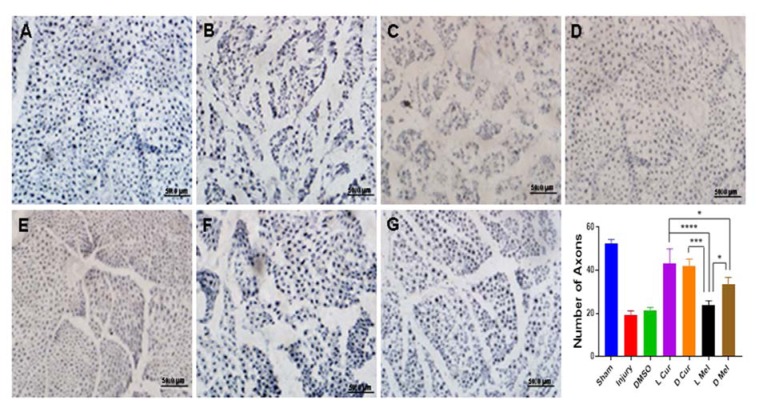
Immunohistochemical staining and quantification of anti-S100. All samples were subjected to Immunohistochemistry staining anti S100 for displaying the number of Schwann cells. The highest number of Schwann cells were found in the sham group (A). No significant difference was observed between injury (B), and DMSO (C) groups and they had the lowest number of Schwann cells. In the treatment groups, no significant difference was found between the light (D) and dark (E) period curcumin groups although curcumin groups showed better results in comparison with melatonin groups. No statistically significant differences were found between L Mel (F), and D Mel (G) groups. * P < 0.05., *** P < 0.001, **** P < 0.0001. Scale bar: 50 µm.

Applying H&E method, Sham, Injury, DMSO, and treatment groups were compared in terms of gastrocnemius muscle fibers diameter. Statistically, no significant difference was found between L Cur and D Cur groups while curcumin groups displayed better results compared with melatonin groups. In the case of melatonin groups, a significant difference was found between D Mel and L Mel groups while data showed that administering melatonin in the dark period could leave a stronger effect. DMSO and injury groups showed no difference and they had the smallest muscle fiber diameter compared with other groups (Figure 3(Fig. 3)). Nerve myelin was assessed using LFB staining. All groups were compared in terms of color intensity. In the case of the sham group, the color intensity was found to be higher than that of other groups. In the treatment groups, L Cur and D Cur groups showed no statistically significant difference; in addition, no difference was found between these groups and sham group. The color intensity was found to be higher in curcumin groups than in melatonin groups. In this regard, the D Mel group proved to be significantly higher than L Mel group. No statistically significant differences were found between injury and DMSO groups. Besides, these groups showed the lowest color density among all groups (Figure 4(Fig. 4)). Immunohistochemistry staining was performed to investigate the existence of Schwann cells and neurofilaments. Data analysis showed that the greatest number of Schwann cells were found in the sham group. A statistically significant difference was observed between sham group and others. No significant difference was observed between DMSO and injury groups and they had the lowest number of Schwann cells. In the case of treatment groups, no significant difference was found between curcumin groups although curcumin groups showed better results in comparison with melatonin groups. No statistically significant differences were found between D Mel and L Mel groups (Figure 5(Fig. 5)).

**Figure 6:**
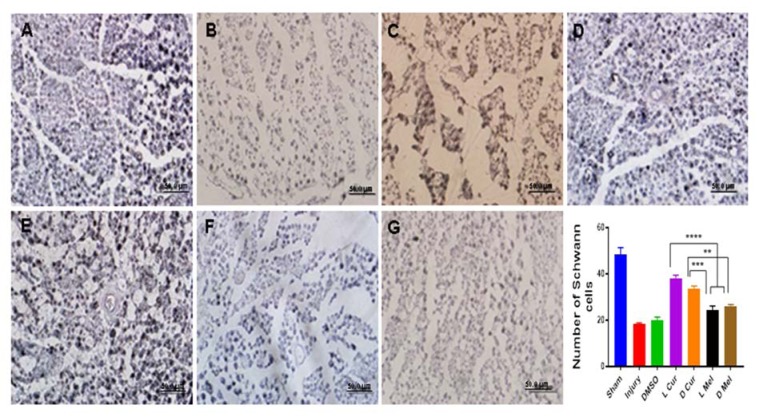
Immunohistochemical staining and quantification of anti NF-200. After immunostaining, all samples were investigated for the number of anti NF-200 positive areas. The findings demonstrated that the sham group (A) showed the greatest number among all groups. Between the treatment groups, there was no significant difference between L Cur (D) and D Cur (E) groups. In addition, the L Cur group did not significantly differ from the sham group but it was significantly different from melatonin groups. Statistically, D Mel (G) group was better than L Mel (F) group. Injury (B) and DMSO (C) groups showed the lowest number. ** P < 0.01, *** P < 0.001, **** P < 0. 0001. Scale bar: 50 µm.

The findings from immunohistochemistry showed greater intense positive staining for NF-200 in the cross-sections of regenerated nerve segments. In this respect, the sham group showed the best result among all groups. In the case of treatment groups, there was no significant difference between L Cur and D Cur groups. Besides, the L Cur group did not significantly differ from the sham group but it was significantly different from melatonin groups. Statistically, D Mel group was better than L Mel group. Injury and DMSO groups showed no statistically significant differences (Figure 6(Fig. 6)).

See also the Supplementary material.

## Discussion

In contrast to the nerves in the central nervous system, peripheral nerves can regenerate after being damaged. When crush injuries happen, axonotmesis may occur in nerves. It should be noted that endoneurial sheath integrity is maintained after axonotmesis but axon destruction and Wallerian degeneration can be observed at the site of injury. Nervous cells death happens after these events and may subsequently lead to injury and end-organ atrophy that is dependent on denervation prolongation and inappropriately directed axonal extension. In addition, scar tissue that develops on site of the injury mechanically prevents axonal extension and as such may affect recovery adversely. Interruption of mechanical transmission and microvasculature in nerve may occur in crush injuries (Sayad Fathi and Zaminy, 2017[38]). As a result, reperfusion leads to pooling of oxygen and nutrients that result in the formation of free radicals. These free radicals possess destructive effects on tissue. Many promising studies have been conducted that involved antioxidant and anti-inflammatory agents while some of these agents have begun to be used in the treating peripheral nerve regeneration. In all these studies, different biological substances are added exogenously to the microenvironment near the damaged axon and are known to affect nerve regeneration at a cellular level (Yüce et al., 2015[45]).

In the present study, melatonin and curcumin were employed as two neuro-protective, antioxidant, and anti-inflammatory drugs for sciatic nerve crush injury in rats. The light and dark periods were compared in relation to their effects. As one of the most common experimental models for peripheral nerve injury, sciatic nerve crush in rodents is commonly employed to evaluate the neuro-regenerative strength of putative drugs (Caillaud et al., 2018[4]). 

Three commonly employed classes of measures (electrophysiology, histomorphometry, and functional tests) have been used to experimentally quantify nerve regeneration. Our data analysis showed that the dark melatonin can yield better results among melatonin groups but no difference was observed among curcumin groups. Following previously reported studies, small doses were selected (Zhao et al., 2017[48]; Sang et al., 2018[37]; Yüce et al., 2015[45]; Zangiabadi et al., 2011[46]; Chang et al., 2014[6]; Lee et al., 2014[19]). 

A derivative of Tryptophan, melatonin is an important signal molecule with wide natural distribution and with a broad range of biological functions: the regulation of the immune system, neuroprotection, antioxidant, anti-inflammation, oncostatic, regulation of pituitary hormones as well as regulatory effects on circadian and endocrine rhythms (Majidinia et al., 2018[23]). The beneficial effects of melatonin on peripheral nerve injury have been indicated in several studies. In addition, melatonin plays an active role in healing nerve injury due to its post-traumatic axon sprouting, broad-spectrum antioxidant properties and also its ability as a powerful inhibitor of apoptosis. From another perspective, the regeneration process depends on the balance between Schwann cell proliferation, on one hand, and scar tissue formation on the other. Therefore, melatonin is an alternative agent that can be used to prevent scar formation surrounding the damaged area by preventing the production of collagen. This signal molecule can also mitigate the myelin damage and axonal alterations in the peripheral nerve. Moreover, according to one study, melatonin also plays a significant role in healing severe sciatic nerve injury through plasmalemma fusion (Altunkaynak et al., 2017[1]). 

However, it has been found that the performance of many therapeutic drugs can be affected by the circadian rest-activity cycle. It has been suggested that administering drugs at certain times of the day could contribute to pharmacotherapy and influence drug efficacy and safety (Ohdo, 2007[30]; Kosobud et al., 2007[18]).

Melatonin is one of the most important drugs with these capabilities. For instance, an experimental study explored the effects of melatonin on sciatic nerve recovery after injury and tried to determine its effects when administered during light and dark periods. In the group treated with melatonin in darkness, it was observed that SFI reached the control level while both muscle contraction and IL-1B were significantly improved. It was concluded that melatonin can enhance neural recovery and stimulate nerve regeneration when rats are treated during the dark period (Rateb et al., 2017[34]). Following these studies, our data also demonstrated that exogenous melatonin can have a stronger effect on sciatic nerve regeneration when administered during the dark period. This could be explained by the fact that melatonin's physiological concentration cannot be sufficient after injury but exogenous melatonin doses greater than 0.5mg possess pharmacologically therapeutic effects (Altunkaynak et al., 2017[1]). When administered during the dark period, melatonin plasma concentration is 3 to 10 times greater than during the light period (Odaci and Kaplan, 2009[29]). Nightly melatonin administration is added to the nocturnal concentration; thus, total concentration and, consequently, its effect are reinforced.

This study has also explored another component: curcumin. Herbal drugs are distinguished from synthetic drugs because of their distinctive characteristics. In addition, chemical synthetic drugs are characterized by a variety of limitations. For one thing, synthetic drugs have side effects and are costly and rarely available in all countries. On the other hand, medicinal herbs have proved beneficial and are gaining importance in the prevention and treatment of inflammatory diseases. Being effective, cheap, and easily available, herbal drugs are increasingly popular. They contain more than one active compound while the active principle is mostly obscure (Yatoo et al., 2018[44]; Sahoo et al., 2010[36]).

Curcumin (1,7-bis (4-hydroxy-3-methoxyphenyl)-1,6-heptadiene-3,5-dione) is the active ingredient in turmeric, one of the most effective herbs in nature. The biological effects and molecular mechanisms of curcumin have been explained in many epidemiological, clinical, and animal studies which demonstrated its anticarcinogenic, antioxidant, antimicrobial, anti-inflammatory, wound-healing, neuroprotective, antimutagen, and angiogenesis regulatory properties (Yüce et al., 2015[45]).

In addition, curcumin can be used to protect the peripheral nerve structure in case of peripheral nerve injury. As the existing studies show, the neuroprotective effect of curcumin for the peripheral nervous system has been widely recognized. Accordingly, curcumin can be used to reduce apoptosis of Schwann cells and promote injury-related cell autophagy, remyelination and axon regeneration in the sciatic nerve of rats (Zhao et al., 2017[48]). Further, another study suggested that curcumin is effective in repairing the entire sciatic nerve injury and that the underlying mechanism may have to do with upregulation of S100 expression as an indicator of Schwann cells proliferation (Liu et al., 2016[21]). In an experimental study, the histological changes in dorsal root ganglion (DRG) and sciatic nerve were quantified in rats that underwent sciatic nerve crush followed by curcumin treatment. It reported that curcumin possesses a protective effect on the DRG and sciatic nerve structure in injured rats (Noorafshan et al., 2011[28]). 

A variety of naturally occurring bioactive molecules (e.g. curcumin) has been reported as an effective antioxidant and anti-inflammatory agents (Ghosh et al., 2015[11]). Curcumin can be used to target several molecular pathways without any risk of toxicity or resistance. The molecular basis for the pleiotropic effects of curcumin has to do with the modulation of various signal molecules (Prasad et al., 2014[33]). A study compared a curcumin-treated diabetic group with an untreated diabetic group and reported that total oxidant status, total antioxidant status, oxidative stress index levels in the brain and sciatic nerve tissues in curcumin group were significantly lesser than in the untreated group (Sahin et al., 2012[35]). On the other hand, curcumin is a unique antioxidant that can be used to protect biomembranes against peroxidative damage. Peroxidation of lipids is known to be a chain reaction mediated by free-radicals that results in damage to cell membranes. Inhibition of peroxidation by curcumin is largely ascribed to the scavenging of the reactive free radicals that are involved in peroxidation (Menon and Sudheer, 2007[26]). Curcumin's scavenging of oxygen radical has been associated with its anti-inflammatory effects (Singh and Vinayak, 2015[40]). Curcumin is also known to be great powerful in anti-inflammatory response (Menon and Sudheer, 2007[26]). The anti-inflammatory properties of polyphenols such as curcumin are well-established that is due to interaction with different cytokines and inflammatory cells like TNF-α, IL-1β, IL-6, and macrophages in different pathological conditions (Caillaud et al., 2018[4]; Singh and Vinayak, 2015[40]). As reported in a recent study composed of several *in vitro *experiments, curcumin can be used to decrease the production of monocyte chemoattractant protein-1 (MCP-1), one of the chief chemokines that regulate migration and tissue infiltration of monocytes/macrophages (Caillaud et al., 2018[4]). Additionally, the natural anti-inflammatory activity of curcumin equals the steroidal and nonsteroidal drugs that are known to have undesirable side effects (Menon and Sudheer, 2007[26]). 

In brief, this study demonstrated the beneficial effects of melatonin and curcumin on nerve regeneration after peripheral nerve crush injury. According to the results, better nerve regeneration was induced by melatonin when rats received treatment in the dark period. However, curcumin's effects did not correlate with the injection time, but curcumin displayed better performance than melatonin in stimulating nerve regeneration with the doses mentioned above. It is suggested that future researches compare melatonin and curcumin effects against those of different nerve-regenerating agents and explore their effectiveness with respect to human nerve injury. 

## Acknowledgements

This research was fully supported by Vice-Chancellor for Research and Technology of Guilan University of Medical Sciences (Grant number: 96061510). This paper is extracted from a master’s thesis project.

## Conflict of interest

Authors declare no conflict of interest for this paper.

## Supplementary Material

Supplementary material
