# Supplementary material for: Comparison of melatonin and curcumin effect at the light and dark periods on regeneration of sciatic nerve crush injury in rats
Source: EXCLI J. 2019 Aug 21;18:653–65. doi: 10.17179/excli2019-1369 (PMC6785766; doi:10.17179/excli2019-1369)
Supplement: Supplementary material [file EXCLI-18-653-s-001.pdf]

EXCLI Journal 2019;18:653-665 – ISSN 1611-2156
Received: April 23, 2019, accepted: August 14, 2019, published: August 21, 2019

Supplementary material to:

COMPARISON OF MELATONIN AND CURCUMIN EFFECT AT THE LIGHT AND DARK PERIODS ON REGENERATION OF SCIATIC NERVE CRUSH INJURY IN RATS

Farshad Moharrami Kasmaie[1], Zohreh Jahromi[1], Rouhollah Gazor[2], Arash Zaminy[3*]

[1] Student Research Committee, School of Medicine, Guilan University of Medical Sciences, Rasht, Iran
[2] Department of Anatomical Sciences, School of Medicine, Guilan University of Medical Sciences, Rasht, Iran
[3] Neuroscience Research Center, Guilan University of Medical Sciences, Rasht, Iran

* Corresponding author: Arash Zaminy, Ph.D. Neuroscience Research Center, Guilan University of Medical Sciences, Rasht, Iran. Neuroscience Research Center, Poursina Hospital, Namjoo Street, Rasht, Iran. P.O. Box: 4193713194, Phone: +981333311472, Fax: +1333368773-98, Cell: +989124796418, E-mail: zaminy_a@gums.ac.ir

http://dx.doi.org/10.17179/excli2019-1369

This is an Open Access article distributed under the terms of the Creative Commons Attribution License (http://creativecommons.org/licenses/by/4.0/).

EXCLI Journal 2019;18:653-665 – ISSN 1611-2156
Received: April 23, 2019, accepted: August 14, 2019, published: August 21, 2019

Supplementary Table 1: Sciatic Functional Index (SFI)

Rat 8	Rat 7	Rat 6	Rat 5	Rat 4	Rat 3	Rat 2	Rat 1	Days post-operation
**Sham**								
-1.97	-3.56	-0.50	-0.83	-2.12	-2.95	-1.91	-3.24	**1**
-4.25	-2.40	-1.21	-3.20	-0.69	-2.85	-1.62	-3.52	**7**
-2.51	-0.46	-1.93	-0.82	-3.58	-0.21	-0.36	-2.14	**14**
-3.36	-1.54	-2.16	-1.50	-2.43	-4.23	-2.16	-1.14	**21**
-2.86	-0.51	-2.43	-1.96	-1.02	-3.24	-0.86	-2.39	**28**
**Injury**								
**Rat 8**	**Rat 7**	**Rat 6**	**Rat 5**	**Rat 4**	**Rat 3**	**Rat 2**	**Rat 1**	**Days post-operation**
-56.50	-49.99	-78.28	-83.14	- 60.19	-82.44	- 75.50	-59.60	**1**
-46.66	-59.20	-70.12	-60.65	-68.25	-75.35	-61.86	-45.63	**7**
-54.04	-71.64	-65.55	-59.55	-32.89	-68.11	-60.50	-44.15	**14**
-62.14	-64.32	-50.01	-69.14	-35.55	-61.11	-54.77	-50.05	**21**
-71.36	-50.10	-45.50	-75.91	-69.13	-53.33	-59.20	-62.87	**28**
**DMSO**								
**Rat 8**	**Rat 7**	**Rat 6**	**Rat 5**	**Rat 4**	**Rat 3**	**Rat 2**	**Rat 1**	**Days post-operation**
-65.23	-73.33	-76.88	-60.87	-69.27	-68.37	-71.23	-61.68	**1**
-66.14	-58.59	-62.37	-54.08	-63.31	-65.02	-66.54	-70.06	**7**
-50.12	-61.30	-55.64	-51.01	-59.45	-52.30	-60.21	-61.05	**14**
-57.14	-55.14	-41.94	-61.01	-60.08	-48.88	-60.03	-51.12	**21**
-64.21	-48.97	-61.07	-50.42	-64.19	-49.13	-61.12	-58.23	**28**
**L Cur**								
**Rat 8**	**Rat 7**	**Rat 6**	**Rat 5**	**Rat 4**	**Rat 3**	**Rat 2**	**Rat 1**	**Days post-operation**
-53.55	-82.18	-43.47	-59.31	-65.04	-62.62	-87.70	-35.19	**1**
-64.53	-58.62	-67.00	-34.36	-64.25	-41.08	-44.92	-36.94	**7**
-10.72	-41.99	-25.14	-33.03	-32.38	-31.41	-18.64	-20.28	**14**
-10.24	-20.09	-23.58	-22.40	-18.85	-28.24	-16.35	-18.77	**21**
-3.69	-9.99	-6.24	-11.20	-8.31	-5.60	-3.56	-6.27	**28**

EXCLI Journal 2019;18:653-665 – ISSN 1611-2156
Received: April 23, 2019, accepted: August 14, 2019, published: August 21, 2019

Supplementary Table 1 (cont.): Sciatic Functional Index (SFI)

D Cur								
Rat 8	Rat 7	Rat 6	Rat 5	Rat 4	Rat 3	Rat 2	Rat 1	Days post-operation
-67.22	-79.08	-46.57	-28.15	-62.15	-53.09	-73.31	-38.16	1
-31.08	-58.10	-51.45	-48.60	-20.85	-41.56	-62.44	-55.46	7
-20.05	-32.11	-64.67	-47.70	-17.08	-27.09	-39.27	-37.50	14
-17.30	-21.32	-34.56	-33.27	-22.41	-28.70	-27.19	-48.23	21
-8.37	-10.18	-9.90	-11.54	-7.63	-12.65	-6.96	-17.23	28
**L Mel**								
Rat 8	Rat 7	Rat 6	Rat 5	Rat 4	Rat 3	Rat 2	Rat 1	Days post-operation
-58.31	-66.49	-72.04	-53.79	-80.71	-56.72	-70.83	-61.03	1
-63.05	-58.54	-69.63	-47.57	-70.23	-55.23	-51.75	-71.79	7
-47.48	-54.89	-68.23	-48.60	-65.89	-50.23	-56.86	-70.23	14
-45.02	-46.21	-60.21	-50.72	-62.24	-44.91	-40.08	-63.08	21
-35.50	-43.25	-41.29	-41.11	-49.30	-48.57	-39.31	-50.63	28
**D Mel**								
Rat 8	Rat 7	Rat 6	Rat 5	Rat 4	Rat 3	Rat 2	Rat 1	Days post-operation
-53.59	-81.21	-60.61	-89.16	-49.63	-57.11	-24.28	-91.07	1
-73.69	-73.25	-67.42	-51.25	-67.25	-49.88	71.64-	-63.92	7
-49.25	-48.39	-42.96	-43.65	-40.02	-29.54	-54.40	-33.84	14
-35.50	-51.66	-24.86	-23.83	-38.50	-37.85	-23.08	-11.35	21
-32.85	-23.56	-29.36	-27.59	-36.37	-10.56	-28.66	-18.35	28

The SFI test was performed on days 1, 7, 14, 21, and 28 following the surgery to show motor functional recovery (n=8). Among treatment groups, the SFI value in curcumin groups was better than Mel groups. In melatonin groups, D Mel group showed a significant difference compared with L Mel group. The above data has been presented in Figure 1A at the main manuscript.

Sham group = surgery without injury
Injury group = crush injury without injection
DMSO (Dimethyl Sulfoxide) = vehicle group
L Cur = Light Curcumin (curcumin administration in light period)
D Cur = Dark Curcumin (curcumin administration in dark period)
L Mel = Light Melatonin (melatonin administration in light period)
D Mel = Dark Melatonin (melatonin administration in dark period)

EXCLI Journal 2019;18:653-665 – ISSN 1611-2156
Received: April 23, 2019, accepted: August 14, 2019, published: August 21, 2019

Supplementary Table 2: Gastrocnemius muscle mass

	Rat 1			Rat 2			Rat 3			Rat 4			Rat 5			Rat 6			Rat 7			Rat 8		
	I	N	M	I	N	M	I	N	M	I	N	M	I	N	M	I	N	M	I	N	M	I	N	M
Sham	1.9	1.9	100	1.53	1.56	98.07	1.8	1.82	98.9	2	2	100	1.55	1.61	96.27	1.7	1.71	99.41	1.89	1.93	97.92	1.95	1.96	99.48
Injury	0.83	2.3	36.08	0.69	1.85	37.29	0.53	1.94	27.31	0.71	2	35.5	0.68	1.98	34.34	0.56	1.89	29.62	0.49	1.98	4.742	0.5	1.8	27.77
DMSO	0.69	1.82	37.91	0.41	1.69	24.26	0.83	2.14	38.78	0.56	1.71	32.74	0.74	1.91	38.74	0.91	2.21	41.17	0.73	1.98	36.86	0.66	1.67	39.52
L Cur	1.49	2.1	70.95	1.51	2	75.5	1.29	1.68	76.78	1.24	1.78	69.66	1.57	1.98	79.2	1.63	1.93	84.4	1.34	1.83	73.22	0.81	1.15	70.43
D Cur	1.54	1.93	79.79	1.37	2.02	67.67	1.69	2.1	80.47	1.18	1.86	63.4	1.28	1.77	72.31	1.1	1.6	68.75	1.2	1.5	80	1.44	1.72	83.72
L Mel	0.52	1.36	38.23	0.71	1.6	44.37	0.69	1.57	43.94	0.77	1.46	52.74	0.79	1.51	52.31	0.69	1.57	43.94	1.03	2.19	47.03	0.7	1.4	50
D Mel	1.03	1.7	60.58	1.02	1.7	60	1.06	1.6	66.38	0.96	1.63	58.89	1.15	2.09	55.02	0.88	1.45	60.68	0.81	1.46	55.47	0.78	1.32	59.09

I = Injured leg
N = Normal leg
M = Muscle Mass (%)

The weight ratio of the gastrocnemius muscle was used in order to perform a recovery assessment on the 28th day after surgery in all groups (n=8). In order to determine the weight ratios (in percentages), the weights of muscle mass from the injured sides were divided by those from the normal sides. The curcumin groups displayed the minimum extent of atrophy and better recovery than melatonin groups. The D Mel group also displayed better results compared with the L Mel group. The mentioned data has been shown in Figure 1 B of the main manuscript.

Sham group = surgery without injury
Injury group = crush injury without injection
DMSO (Dimethyl Sulfoxide) = vehicle group
L Cur = Light Curcumin (curcumin administration in light period)
D Cur = Dark Curcumin (curcumin administration in dark period)
L Mel = Light Melatonin (melatonin administration in light period)
D Mel = Dark Melatonin (melatonin administration in dark period)

EXCLI Journal 2019;18:653-665 – ISSN 1611-2156
Received: April 23, 2019, accepted: August 14, 2019, published: August 21, 2019

Supplementary Table 3: Electrophysiological test

	Rat 1		Rat 2		Rat 3	
	Late	Amp	Late	Amp	Late	Amp
Sham	0.6	42.3	0.9	61.5	1	39.8
Injury	2.7	3.1	2.7	1.5	2.7	0.5
DMSO	2.5	0.9	2.9	2.6	2.3	1.6
L Cur	1.1	39.5	0.9	36.4	1.3	49.3
D Cur	1.4	31.2	1.3	28.8	1.1	47.6
L Mel	1.8	22.5	1.7	1.7	2	15.2
D Mel	1.7	16.1	2	5.3	1.7	11.4

On the 28th post-operation day, motor functional recovery was assessed using electrophysiological test (n=3). The onset latency and peak amplitude of CMAP were analyzed for all groups. The L Cur and D Cur groups showed no significant difference and, compared with other groups, they had the least latency and the greatest amplitude. The above table has been presented as Figure 1C-D of the main manuscript.

Amp = amplitude
Late = latency
Sham group = surgery without injury
Injury group = crush injury without injection
DMSO (Dimethyl Sulfoxide) = vehicle group
L Cur = Light Curcumin (curcumin administration in light period)
D Cur = Dark Curcumin (curcumin administration in dark period)
L Mel = Light Melatonin (melatonin administration in light period)
D Mel = Dark Melatonin (melatonin administration in dark period)

EXCLI Journal 2019;18:653-665 – ISSN 1611-2156
Received: April 23, 2019, accepted: August 14, 2019, published: August 21, 2019

Supplementary Table 4: Total Oxidant Status (TOS)

	Rat 1	Rat 2	Rat 3
Sham	0.171	0.174	0.163
Injury	0.197	0.193	0.193
DMSO	0.186	0.181	0.196
L Cur	0.101	0.107	0.119
D Cur	0.1	0.129	0.114
L Mel	0.136	0.18	0.199
D Mel	0.153	0.146	0.193

The total amount of oxidants for all serum samples was checked on the 28th day after surgery in all groups (n=3). Values are given as mean. In curcumin groups, TOS was significantly lesser than melatonin groups. No statistically significant difference was found between L and D Mel groups. The above table has been demonstrated in Figure 2 of the main manuscript.

Sham group = surgery without injury
Injury group = crush injury without injection
DMSO (Dimethyl Sulfoxide) = vehicle group
L Cur = Light Curcumin (curcumin administration in light period)
D Cur = Dark Curcumin (curcumin administration in dark period)
L Mel = Light Melatonin (melatonin administration in light period)
D Mel = Dark Melatonin (melatonin administration in dark period)

EXCLI Journal 2019;18:653-665 – ISSN 1611-2156
Received: April 23, 2019, accepted: August 14, 2019, published: August 21, 2019

Supplementary Table 5: Hematoxylin & Eosin staining

	Rat 1	Rat 2	Rat 3
**Sham**	66.13124	65.91464	60.80704
**Injury**	24.76339	23.29668	22.21747
**DMSO**	25.98123	37.68798	24.4881
**L Cur**	63.81726	50.88696	55.57109
**D Cur**	52.73889	53.36015	50.54598
**L Mel**	33.49252	35.78711	28.08867
**D Mel**	43.24690254	45.52523954	45.65429886

Hematoxylin-Eosin staining (H&E) was used to show the morphology of the gastrocnemius muscle and to determine the diameter of muscle fibers on 28th day after surgery. Statistically, curcumin groups displayed better results compared with melatonin groups. In melatonin groups, a significant difference was found between D Mel and L Mel groups while data showed that administering melatonin in the dark period left a stronger effect. The mentioned data has been shown as Figure 3 of the main manuscript.

Sham group = surgery without injury
Injury group = crush injury without injection
DMSO (Dimethyl Sulfoxide) = vehicle group
L Cur = Light Curcumin (curcumin administration in light period)
D Cur = Dark Curcumin (curcumin administration in dark period)
L Mel = Light Melatonin (melatonin administration in light period)
D Mel = Dark Melatonin (melatonin administration in dark period)

Received: April 23, 2019, accepted: August 14, 2019, published: August 21, 2019

Supplementary Table 6: Luxol fast blue staining

	Rat 1	Rat 2	Rat 3
**Sham**	178.7593	177.1553	176.711
**Injury**	155.5743	149.193	148.6863
**DMSO**	150.9083	157.611	159.5227
**L Cur**	174.2097	173.796	174.3533
**D Cur**	172.1333	174.3027	173.535
**L Mel**	156.2027	161.0803	155.6347
**D Mel**	165.886	163.2043	167.2427

Myelin content was evaluated by Luxol Fast Blue (LFB) staining on 28th day after surgery. The color intensity in curcumin groups was found higher than in melatonin groups. The D Mel group proved to be significantly lower than the L Mel group. The above data has been shown as Figure 4 of the main manuscript.

Sham group = surgery without injury
Injury group = crush injury without injection
DMSO (Dimethyl Sulfoxide) = vehicle group
L Cur = Light Curcumin (curcumin administration in light period)
D Cur = Dark Curcumin (curcumin administration in dark period)
L Mel = Light Melatonin (melatonin administration in light period)
D Mel = Dark Melatonin (melatonin administration in dark period)

EXCLI Journal 2019;18:653-665 – ISSN 1611-2156
Received: April 23, 2019, accepted: August 14, 2019, published: August 21, 2019

Supplementary Table 7: Immunohistochemistry: Anti-S100

	Rat 1	Rat 2	Rat 3
**Sham**	47.46667	51.73333	45.93333
**Injury**	18.53333	18.73333	17.6
**DMSO**	18.86667	19.6	21.6
**L Cur**	38.06667	39.4	36.33333
**D Cur**	33.73333	34.73333	32.2
**L Mel**	22.53333	23.93333	26.33333
**D Mel**	25.6	26.53333	26.53333

Using the light microscope, Anti S100 immunoreactivity was clearly observed to investigate the existence of Schwann cells. In treatment groups, no significant difference was found between curcumin groups. However, curcumin groups showed better results in comparison with melatonin groups. No statistically significant differences were found between D Mel and L Mel groups. The aforementioned data has been shown as Figure 5 of the main manuscript.

Sham group = surgery without injury
Injury group = crush injury without injection
DMSO (Dimethyl Sulfoxide) = vehicle group
L Cur = Light Curcumin (curcumin administration in light period)
D Cur = Dark Curcumin (curcumin administration in dark period)
L Mel = Light Melatonin (melatonin administration in light period)
D Mel = Dark Melatonin (melatonin administration in dark period)

EXCLI Journal 2019;18:653-665 – ISSN 1611-2156
Received: April 23, 2019, accepted: August 14, 2019, published: August 21, 2019

Supplementary Table 8: Immunohistochemistry: Anti neurofilaments-200

	Rat 1	Rat 2	Rat 3
**Sham**	51.93333	54.53333	51.2
**Injury**	20	16.93333	20.66667
**DMSO**	22.6	21.6	19.73333
**L Cur**	45.07	48.86666667	36.06666667
**D Cur**	38.86666667	45.66666667	40.6
**L Mel**	24.6	25.46666667	21.73333
**D Mel**	34.73333	36.13333	30.26667

The morphometric analyses of regenerated nerves for each of the experimental groups on the 28th day after surgery. The findings from immunohistochemistry showed that there was greater intense positive staining for NF-200 in the cross sections of regenerated nerve segments. In treatment groups, there was no significant difference between L Cur and D cur groups. As well as the L Cur group was significantly different from melatonin groups. Statistically, D Mel group was better than L Mel group. The data has been shown as Figure 6 of the main manuscript.

Sham group = surgery without injury
Injury group = crush injury without injection
DMSO (Dimethyl Sulfoxide) = vehicle group
L Cur = Light Curcumin (curcumin administration in light period)
D Cur = Dark Curcumin (curcumin administration in dark period)
L Mel = Light Melatonin (melatonin administration in light period)
D Mel = Dark Melatonin (melatonin administration in dark period)
